# Does Cannabis, Cocaine and Alcohol Use Impact Differently on Adult Attention Deficit/Hyperactivity Disorder Clinical Picture?

**DOI:** 10.3390/jcm10071481

**Published:** 2021-04-02

**Authors:** Vincenza Spera, Alessandro Pallucchini, Marco Carli, Marco Maiello, Angelo G. I. Maremmani, Giulio Perugi, Icro Maremmani

**Affiliations:** 1 Psychiatric Clinic, Sociopsychiatric Organization, 6850 Mendrisio, Switzerland; e.spera@hotmail.it; 2 PISA-School of Experimental and Clinical Psychiatry, 56100 Pisa, Italy; pallucchini.a@gmail.com (A.P.); marcomaiello@aol.com (M.M.); angelo.maremmani@uslnordovest.toscana.it (A.G.I.M.); 3 School of Clinical Pharmacology, Department of Clinical and Experimental Medicine, University of Pisa, 56100 Pisa, Italy; carlimarco@outlook.it; 4 North-Western Tuscany Local Health Unit, Department of Psychiatry, Tuscany NHS, Versilia Zone, 55045 Viareggio, Italy; 5 Association for the Application of Neuroscientific Knowledge to Social Aims (AU-CNS), Pietrasanta, 55045 Lucca, Italy; 6 2nd Psychiatric Unit, Department of Clinical and Experimental Medicine, Santa Chiara University Hospital, University of Pisa, 56100 Pisa, Italy; giulio.perugi@med.unipi.it; 7 Vincent P. Dole Dual Disorder Unit, 2nd Psychiatric Unit, Santa Chiara University Hospital, University of Pisa, 56100 Pisa, Italy; 8 G. De Lisio Institute of Behavioral Sciences, 56121 Pisa, Italy; 9 Saint Camillus International University of Health and Medical Sciences—UniCamillus, 00131 Rome, Italy

**Keywords:** attention deficit hyperactivity disorder, adult ADHD, substance use disorder, dual disorder

## Abstract

While the association between adult Attention Deficit/Hyperactivity Disorder (A-ADHD) and Substance Use Disorders (SUDs) has been widely explored, less attention has been dedicated to the various substance use variants. In a previous paper, we identified two variants: type 1 (use of stimulants/alcohol) and type 2 (use of cannabinoids). In this study, we compared demographic, clinical and symptomatologic features between Dual Disorder A-ADHD (DD/A-ADHD) patients according to our substance use typology, and A-ADHD without DD (NDD/A-ADHD) ones. NDD patients were more frequently diagnosed as belonging to inattentive ADHD subtype compared with type 1 DD/A-ADHD patients, but not with respect to type 2 DD/ADHD. NDD/A-ADHD patients showed less severe symptoms of hyperactivity/impulsivity than DD/A-ADHD type 1, but not type 2. Type 1 and type 2 patients shared the feature of displaying higher impulsiveness than NDD/A-ADHD ones. General psychopathology scores were more severe in type 2 DD/ADHD patients, whereas type 1 patients showed greater similarity to NDD/A-ADHD. Legal problems were more strongly represented in type 1 than in type 2 patients or NDD/A-ADHD ones. Our results suggest that type 1 and type 2 substance use differ in their effects on A-ADHD patients—an outcome that brings with it different likely implications in dealing with the diagnostic and therapeutic processes.

## 1. Introduction

Attention Deficit Hyperactivity Disorder (ADHD), which was originally identified as a childhood neuropsychiatric illness, is now known to endure into adulthood in about two-thirds of these patients, with adult prevalence rates estimated to be close to the 3–5% range. Even if it has been defined as “a persistent pattern of inattention and/or hyperactivity-impulsivity that interferes with functioning or development” (Diagnostic and Statistical Manual of Mental Disorders, 5th edition (DSM-5)) [1], in adults, ADHD (A-ADHD) reflects a syndrome featuring mood instability, Emotional Dysregulation, impulsiveness and addictive behaviours [2]. The comorbidity of ADHD and Substance Use Disorders (SUD) has been widely documented and recognized in research studies, and the co-occurrence of the two disorders can be defined as Dual Disorder (DD). Indeed, the term Dual Disorders refers to patients with both a Substance Use Disorder and a co-occurring mental one. ADHD and SUD are often associated since they share common genetic underpinnings, neurobiological substrates, and risk factors [3,4,5]. A metanalysis showed an ADHD prevalence of 23.1% among SUD population [6]; similarly, a multicenter study of treatment seeking SUD patients revealed a 13.9% prevalence of adult ADHD [7]. The predisposition to addictive behaviours and SUD that is often found in ADHD patients could be explained by reference to several mechanisms. Subjects with ADHD usually report higher levels of “sensation-seeking” traits and impulsiveness that invariably activate psychosocial risk factors, such as educational failures and interpersonal problems, and having been exposed to an earlier contact with addictive drugs [8]. Furthermore, the ADHD syndrome and SUD are both impacted by dopaminergic dysregulation of the motivational and reward systems that leads directly to executive dysfunctions and impairment in response inhibition. As a result of dopaminergic dysfunction, some ADHD patients might react by using drugs, especially stimulants, in order to self-medicate, so as to cope with inattention and restlessness symptoms according to the concept of “relief craving” [2]. The “self-medication hypothesis” claims to cover all cases in the same way, but people with ADHD actually rely on a wide variety of illicit substances, among which the best represented are cannabinoids, stimulants and alcohol [9,10,11,12,13]. Overall, in the medical literature, the association of ADHD with SUD configures a worse clinical picture, comprising a more rapid and severe SUD progression [14] in addition to more frequent hospitalizations and a weaker response to treatment [15]. Research data are, however, lacking on the influence of the different substances of abuse on ADHD clinical symptomatology in adult samples.

In a previous study with a sample of 72 patients entering treatment, we identified two patterns of substance use: the first (type 1) centered on the recourse to stimulants/alcohol, and the second (type 2) on the use of cannabinoids (THC). Type 1 DD/A-ADHD was found to occur in significantly younger people, who had to cope with more legal problems than type 2 ones. The two patterns were similar in terms of ADHD-specific symptomatology and its severity at treatment entry. No differences were found with the other scales assessed, except for lower scores in answering the Morningness-Eveningness Questionnaire (MEQ) in type 1 DD/A-ADHD patients. We concluded that, at treatment entry, the presence of different comorbid SUD variants did not affect ADHD-specific symptomatology or the severity of DD/A-ADHD [16].

The aim of the current study was to assess the influence of type 1 and type 2 substance use on the A-ADHD clinical picture by comparing the demographic, clinical, and functional features of patients—those with and without a DD/A-ADHD.

## 2. Materials and Methods

### 2.1. Design of the Study

This was an observational, cross-sectional, non-interventional, retrospective study based on data derived from two Italian psychiatric databases. Patients were recruited from the ADHD Outpatient Clinic of the University of Pisa and from the ADHD Outpatient Clinic of La Sapienza University in Rome. We merged the data, as the two clinics adopted the same sample selection, assessment methods and assessment of clinical features. This study was based on a single evaluation of adult patients admitted between 2016 and 2019. All the patients recruited in the study were evaluated through a clinical interview, self-report questionnaires and scales by residents of the Department of Clinical and Experimental Medicine, School of Psychiatry of the University of Pisa, and by residents of the Psychiatric Unit of La Sapienza University, in Rome, Italy, with at least 2 years of experience in the adult ADHD field, and under the supervision of senior psychiatrists active in the ADHD research group. Patients with schizophrenia spectrum disorders according to DSM-5 criteria were excluded from the clinical sample.

The study was conducted according to the World Medical Association Declaration of Helsinki—Ethical Principles for Medical Research Involving Human Subjects. Both the consent form and the experimental procedures were approved by the ethics committee of the University of Pisa (study ID: 14003; code: ADHD-MOOD), in accordance with internationally accepted criteria for ethical research.

### 2.2. Sample

Out of 183 patients selected, 166 entered the study; five patients were excluded due to schizophrenia comorbidity, and 12 refused to participate due to personal reasons. Among the 166 patients recruited, 113 (68.04%) were males, and 53 (32.0%) were females, mean age 28.30 ± 10.7 (min 18, max 55); of these, 85 were substance non-users (NDD/A-ADHD), 56 (65.9%) males and 29 (34.1%) females, mean age 28.99 ± 11.5; a total of 81 patients, comprising 57 (70.4%) males and 24 (29.6%) females, mean age 27.58 ± 9.8, were also affected by a Substance Use Disorder (Dual Disorder). Both conditions were diagnosed according to the DSM-5 criteria. Using our methodology, [16] 41 DD/A-ADHD patients (of whom 27 (65.9%) were males and 14 (34.1%) females, mean age 28.54 ± 10.2 (min 18, max 51) were diagnosed as type 1, and 40 (comprising 30 (75.0%) and 10 (25.0%) females, mean age 26.60 ± 9.4 (min 18, max 55)) as type 2. All the patients selected were adults (age-range: 18–65 years) whose ADHD was first diagnosed after entry into adulthood (i.e., after reaching the age of 18). Age (F = 0.68; *p* = 0.506) and sex (χ^2^ = 1.23; *p* = 0.583) were homogenous between the three groups being compared. Legal problems were self-reported and included crimes for drug possession or sale, burglary or stealing, aggression or violence related or unrelated to drugs, and driving crimes.

### 2.3. Instruments

In addition to the application of DSM-5 criteria, ADHD diagnosis was further assessed by means of the Adult ADHD Self-Report Scale (ASRS) for screening purposes [17] and confirmed using the Diagnostic Interview for ADHD in adults (DIVA 2.0) [18,19].

The Conners’ Adult ADHD Rating Scales–Observer: Short Version (CAARS-O:S) was completed by a close relative of the patient. This made the quantification of ADHD symptoms and the ADHD Index possible; high scores obtained on the ADHD Index suggest clinically significant levels of ADHD symptoms [20].

Difficulties in the Emotion Regulation Scale (DERS) is a 36-item self-evaluation scale comprising six subdomains of emotional regulation (awareness, clarity, goals, impulse, non-acceptance, strategies). Answers range from 1 (“almost never”) to 5 (“almost always”), and higher total scores recorded in answering DERS reflect greater difficulties in regulating emotions [21].

The Structured Clinical Interview for Axis I Disorders (SCID-I) was used for the assessment of other psychiatric disorders, according to DSM-5 procedures.

The Barratt impulsiveness scale (BIS-11) is a 30-item questionnaire used for the assessment of impulsiveness, which is factorialized into three second-order domains named “Attentional”, “Motor” and “Non-planning” impulsivity [22].

For each patient, the clinician completed the 18-item version of the Brief Psychiatric Rating Scale (BPRS-18)—a tool commonly used to assess general psychopathology. The BPRS explores symptoms typically grouped into five subscales: Anxiety-Depression, Anergia/negative factor, Thought Disturbance, Activation, Hostility-Suspiciousness and Total Psychopathology Severity [23,24].

The Reactivity Intensity Polarity Stability Questionnaire (RIPoSt-40) is a self-report questionnaire comprising 40 items that are used to measure Emotional Dysregulation (ED), which can be further subdivided into four subscales known as “Emotional Impulsivity”, “Positive Emotionality”, “Negative Emotionality” and “Affective Instability” [25].

The World Health Organization Disability Assessment Schedule (WHODAS 2.0), developed directly by the WHO, is a 36-item self-administered questionnaire that is used to explore functioning and disability in major life domains [26].

The 19-item Morningness–Eveningness Questionnaire (MEQ) was used to assess patients’ chronotype: a total score of 41 or below is interpreted as indicating an “evening chronotype”, whereas a score of 59 or above is considered to mark out a “morning chronotype” [27].

### 2.4. Data Analysis

We clustered our patients in different typologies of substance of use by adopting the following procedure. We scaled the use of each substance by defining four ordinal levels: 0 = no lifetime use; 1 = past use (at least six months of abstinence); 2 = current (during the previous 6 months) use; 3 = both past and current use. An exploratory factorial analysis was performed on the substances investigated (cannabinoids, cocaine, amphetamines, 3,4-methylenedioxymethamphetamine or MDMA, opiates, alcohol, illegal benzodiazepines) in ADHD patients, in order to identify possible composite dimensions. The initial factors were extracted by means of principal component analysis (PCA-type 2) and then rotated according to Varimax criteria in order to achieve a simple structure. The criterion used to select the number of factors was an eigenvalue >1. Items loading with absolute values >0.40 were used to describe the factors. This procedure makes it possible to minimize the correlations between the factors, thus allowing their optimization as classificatory tools for each subject. The factor scores were then standardized as z-scores to facilitate the comparison of scores occurring among the factorial measures. All the subjects were then grouped into different subtypes on the basis of the highest z-scores obtained for each factor. This procedure gave the opportunity to classify groups of subjects on the basis of the most statistically abnormal substance use cluster. In this way, it became possible to resolve the problem of identifying a cut-off for the inclusion of patients in different identified clusters. In this specific case, to be able to cluster patients in groups, we used correlations between the substances used.

The three groups (NDD/A-ADHD, DD/A-ADHD Type 1 and DD/A-ADHD Type 2) were compared for sociodemographic, clinical and functional characteristics by means of the chi-square test for categorical variables (with z-test contrasts and Bonferroni’s correction), and one-way analysis of variance for continuous variables, a posteriori contrasts according to the Scheffé’s procedure.

All analyses were carried out using the statistical package of SPSS (version 25.0). Since this is an exploratory study, statistical tests were considered significant at the *p* < 0.05 level.

## 3. Results

Table 1 reports demographic characteristics. No differences were found in investigating age, years of education, sex or civil status. Type 1 DD/A-ADHD patients showed a greater number of legal problems than type 2 and NDD/A-ADHD ones.

Table 2 shows clinical and functional differences. In evaluating ADHD-specific symptomatology by the CAARS-O:S, no differences were found in the scores for the severity of inattentiveness or in the combined scores. The ADHD Index showed similarities between NDD and DD/A-ADHD patients. Conversely, the NDD/A-ADHD group showed less severe hyperactivity/impulsivity symptomatology than in DD/A-ADHD type 1 but not in type 2 patients.

Using DIVA 2.0, NDD patients were found to have been more frequently diagnosed with the inattentive ADHD subtype than type 1 DD/A-ADHD patients, but not with respect to type 2 DD/A-ADHD ones.

No differences were found by comparing difficulties in emotion regulation (DERS) or in its severity (RIPoSt-40); the degree of disability was very similar between the groups.

On symptoms of impulsiveness (BIS-11), type 1 and type 2 patients displayed greater impulsiveness than NDD/A-ADHD ones. General psychopathology (BPRS) scores were more severe in type 2 DD/A-ADHD patients, whereas type 1 showed similarities with NDD/A-ADHD patients.

The presence of other psychiatric comorbidities was not statistically different among the three groups.

## 4. Discussion

In summary, DD/A-ADHD showed greater impulsivity than their peers without DD; more specifically, type 1 and type 2 patients showed the same degree of impulsivity. Type 2 showed more general psychopathology (BPRS) than type 1, while their NDD/A-ADHD peers, interestingly, showed the same degree of psychopathological severity. Type 1, but not type 2 patients, were more hyperactive/impulsive and less often diagnosed as belonging to the inattentive A-ADHD type. Type 1 also showed more frequent legal problems.

On the topic of legal problems, our patients—more specifically, those abusing stimulants and/or alcohol—confirmed data appearing in the literature that show a higher frequency of violent behaviors, together with antisocial and illicit forms of conduct among people with SUD and ADHD [28]. Again, in accordance with previous reports, a majority of our NDD patients at the moment of entry into treatment showed a combined ADHD subtype, with higher frequencies of inattentive symptoms, compared with those found in users of stimulants/alcohol (type 1), and lower impulsivity [29,30].

Regarding our results, general psychopathology could help us discern the effects of different substances on patients with ADHD. Cannabinoid users had a more severe psychopathology, while displaying more activation, hostility, suspiciousness and thought disturbances [31]. In the literature on adult ADHD patients, few studies have paid any attention to the influence of specific substances on ADHD psychopathology, focusing instead on worse all-inclusive outcomes in patients with Dual Disorder arising from substance polyuse and dependence. Our results are not at all surprising, since the use of THC has been widely associated with psychosis, paranoia and aggressiveness in clinical populations, especially in chronic and heavy users [32]. The psychopathological burden seems to be even worse in ADHD individuals, who are more vulnerable to cannabis effects because of the worsening of already present neurocognitive deficits, impulsivity and mood instability.

Even if the association of SUD and ADHD has been widely studied in the literature, few authors have ever examined the role played by specific substances in affecting psychopathological domains. A recent study of ADHD patients seeking treatment for a SUD (consumption of cocaine or cannabis, or both) showed a higher addiction severity in cannabis use groups, with similar ADHD features found between different groups [33]. To the best of our knowledge, no other ADHD study has compared stimulant users with cannabinoid users—to cite the two most frequently abused classes of drugs. The present study did not, in any case, have the aim of comparing two different classes of substance being regularly used.

When using the BIS scale to differentiate between two diverse typologies of substance abuse, the results are unsatisfactory. Both groups show similar, higher scores of impulsiveness compared with NDD/A-ADHD patients. This finding may suggest that, regardless of the substance of abuse, illicit drugs always have a negative impact on the behavior of ADHD patients by enhancing impulsive behavior and loss of self-control. Alternatively, it may be that ADHD patients with higher impulsivity levels at baseline (in youth) are at more risk of developing SUD [34,35].

Our data raise the hypothesis of a distinctive form of substance use in a real-world population of A-ADHD [36]. Users of stimulants might behave in this way to attenuate their symptoms of inattentiveness [37]. If this is true, this new hypothesis might prove to be useful when it comes to selecting patients who do not appear to be inattentive. On the other hand, THC does not mask inattentiveness. According to Khantzian’s “self-medication” hypothesis, ADHD subjects may use stimulants as a way to manage symptoms of impulsivity, restlessness and inattentiveness by obtaining a “calming” effect arising from an increased, specifically dopaminergic transmission (which is dysfunctional in ADHD). The effects of cocaine mainly involve structures that form part of the reward system (accumbens and ventral pallidum), working memory (amygdala and hippocampus), volition (orbitofrontal and subcallosal cortices) and control (prefrontal cortex and cingulate gyrus) [38]. Cocaine inhibits the reuptake of dopamine, norepinephrine and serotonin from the synaptic cleft, enhancing the stimulation mediated by these monoamines [39]. The net effect, in non-ADHD individuals, is a global psychomotor activation involving increased arousal, euphoria and increased vigilance and alertness. Cocaine acts on the systems that appear to be damaged in ADHD patients which are, in fact, the primary target for the pharmaceutical treatment of the disorder. Its use, therefore, may be supported by mechanisms associated with craving for relief, by reducing the burden of executive and behavioral dysfunctions typical of ADHD [40]. Support for this hypothesis can be found in a recent study of ours about psychopathological typology and the severity of symptoms affecting 24 Cocaine Use Disorder (CUD) patients without a Dual Disorder and 120 Cocaine Use Disorder/A-ADHD patients, while assuming that CUD patients are mainly motivated by a craving for reward, whereas Cocaine Use Disorder/A-ADHD patients are mainly driven by a craving for relief [41].

THC acts on the cannabinoid receptors within the brain that are usually responsible for producing affective feelings of well-being, euphoria, reduction of anxiety, cognitive, somatic and sensory effects, such as memory lapses, difficulty in concentration, time distortion, and increased sensitivity to external and internal stimuli [42]. THC is also involved in modulating other neurotransmitters like dopamine, serotonin, glutamate, norepinephrine, γ-aminobutyric acid (GABA), histamine, acetylcholine, prostaglandins and opioid peptides [43]. In particular, its action on the dopaminergic system leads to a reinforcing effect on the conduct of abuse. In addition, through cannabinoid-1-receptors, located in the striatum and substantia nigra, THC elicits striatal dopamine release, thus playing a role in the salience attribution processes by increasing the salience of usually non-salient stimuli [44]. With chronic and prolonged use, the dopaminergic system faces depletion, faltering in an amotivational syndrome and leading to a subsequent impoverishment of executive functions (planning, working memory, etc.) [45]. ADHD patients, therefore, may seek the relaxing and pleasurable effects that cannabis elicits, with a modality we would call reward craving, although the long-term effects ultimately lead to a worsening of ADHD symptomatology [46]. This implies that the first typology of patients (stimulant users) is easier to treat compared with the second typology (THC users), because the initiation of stimulant medications might produce the ending of the harm use of illicit stimulants. Indeed, one of our previous studies showed a reduction in cocaine use correlated with ADHD when stimulant or atomoxetine treatment was initiated [36]. In any case, it should be recognized that all the stimulants, such as cocaine, may trigger impulsivity and foster aggressive behaviours through the increase in dopamine levels in the mesolimbic and mesocortical areas of the brain. Therefore, while the trait of inattentiveness is masked by the use of cocaine, levels of impulsivity prove to be similar in the type 1 and type 2 groups. In general, studies in the literature on the motivation to use substances in individuals with ADHD are controversial, because they suggest both self-therapeutic and euphorigenic use [47]. Research findings regarding treatment options for Dual Disorder patients are inconclusive since stimulant medications—the front-line treatment for ADHD—have mainly shown benefits for ADHD symptomatology, but much less so for SUD. Indeed, Notzon et al. observed an increased abstinence from cannabis in patients with both ADHD and cocaine use disorders after stimulant treatment with extended-release mixed amphetamine salts [48]. Other authors have demonstrated an improvement both in ADHD and cocaine use disorder after treatment with stimulant medications, suggesting a possible role for these specific treatments even when the medical condition in question is SUD [49].

### Clinical Implications

Cocaine, with its prodopaminergic effects, might mask several symptoms of ADHD, and may thus constitute a confounding factor for the correct recognition of the underlying disorder and the definition of a valid strategy for treating a Dual Disorder patient. On the other hand, when impulsivity is indeed present, it becomes necessary to exclude the possibility that it may be due to the SUD. If this is the case, it is important to treat the substance use, and not only the impulsivity, with psychiatric medications. We suggested a hierarchic approach, in which treatment of impulsiveness due to SUD should come first [50,51]. When the treatment aims only to reduce the impulsivity deriving from ADHD, the component due to SUD, without any specific treatment, it may continue to interfere with these patients’ lives and lead to negative outcomes. We believe that people with ADHD suffering from SUD may be driven either by a relief or a reward craving—two different types that may even coexist—especially in advanced illnesses, so providing the preconditions for the worst psychopathological syndromes. The predominance of relief craving during the use of stimulants leads us to hypothesize a better response to specific drugs for ADHD. Conversely, if reward drive is prevalent with the heavy use of cannabinoids, we are often faced by major psychiatric complications, such as mood instability, impulsivity and psychosis.

To our knowledge, our study is the first to analyze patterns of substance use among DD A-ADHD patients and their relationship with the psychopathological outcomes. Although our results are preliminary, and require further research, they may give an important contribution for the evaluation of DD patients with ADHD and their treatment.

Several limitations have to be addressed. First of all, the sample size was not big enough to generalize our findings. All the patients evaluated were never diagnosed or treated for ADHD before adulthood and presented with a severe clinical picture. To confirm our data, a wider sample should be enrolled. The second limitation is the self-assessment of several features, such as impulsivity, Emotional Dysregulation and overall functioning, which may lead to reporting biases. Finally, our sample did not include patients treated exclusively at the “addiction units”; this is especially true for opioid addicts who, indeed, were minimally represented in our sample. In Italy, psychiatric units and addiction units work independently, resulting in poor communication between the two. These sample selection biases limit the generalization of our data.

## 5. Conclusions

Testing the type 1 and type 2 substance modality of use is a useful tool for studying the symptomatological variants of A-ADHD. These variants may be related to the different concepts of craving that are experienced by individuals with a DD/A-ADHD, adding different possible implications in the task of responding to the diagnostic and therapeutic processes.

## Figures and Tables

**Table 1:** Demographic and clinical differences in adult Attention Deficit/Hyperactivity Disorder (A-ADHD) patients with and without Dual Disorder (DD).

	NDD/A-ADHD *N* = 85	DD/A-ADHD Type 1 *N* = 41	DD/A-ADHD Type 2 *N* = 40		
	**M ± SD**	**M ± SD**	**M ± SD**	**F**	***p***
Age	28.99 ± 11.5	28.54 ± 10.2	26.60 ± 9.3	0.68	0.506
Years of education	13.63 ± 2.8	12.78 ± 2.9	13.51 ± 4.1	0.98	0.376
	*N* (%)	*N* (%)	*N* (%)	χ^2^	*p*
Male sex	58 (66.7)	27 (64.3)	30 (75.0)	1.23	0.538
Legal problems	3 (3.5) a	14 (35.0) b	3 (7.5) a	26.34	0.000
Single	65 (78.3)	34 (85.0)	32 (82.1)	0.82	0.661

NDD/A-ADHD = Non-Dual Disorder A-ADHD; DD/A-ADHD = Dual Disorder A-ADHD, M = mean, SD = standard deviation. “a” and “b” letters denotes a subset of categories whose column proportions differ significantly from each other at the 0.05 level.

**Table 2:** Demographic and clinical differences in A-ADHD patients with and without Dual Disorder.

	NDD/A-ADHD *N* = 85	DD/A-ADHD Type 1 *N* = 41	DD/A-ADHD Type 2 *N* = 40		
	**M ± SD**	**M ± SD**	**M ± SD**	**F**	***p***
CAARS-Inattentive	18.08 ± 5.6	16.79 ± 5.3	18.05 ± 5.9	0.82	0.438
CAARS-Hyperactive/Impulsive	12.84 ± 6.5 a	16.00 ± 5.3 b	15.63 ± 6.9 ab	4.69	0.010
CAARS-Combined	30.77 ± 10.6	32.24 ± 9.5	33.55 ± 11.5	1.00	0.369
CAARS-Index	22.98 ± 6.6	22.93 ± 5.0	24.28 ± 6.2	0.69	0.501
Total DERS	2.94 ± 0.5	2.96 ± 0.7	3.01 ± 0.6	1.57	0.855
Total RIPOST	222.12 ± 43.2	219.00 ± 44.4	228.20 ± 48.3	0.39	0.679
Total BIS	75.46 ± 11.7 a	81.60 ± 9.9 b	84.87 ± 12.6 b	10.01	0.000
Total BPRS	38.04 ± 13.5 a	36.43 ± 12.9 a	46.35 ± 16.7 b	6.06	0.003
Total WHODAS	2.45 ± 0.7	2.63 ± 0.6	2.69 ± 0.8	1.74	0.178
	*N* (%)	*N* (%)	*N* (%)	χ^2^	*p*
DIVA combined	57 (67.9)	34 (82.9)	31 (77.5)	3.59	0.166
DIVA inattentive	25 (29.8) a	4 (9.8) b	7 (17.5) ab	7.04	0.030
DIVA hyperactive/impulsive	3 (3.5)	2 (4.9)	2 (5.0)	0.21	0.896
MEQ typology				2.44	0.655
1-Indifferentiate	56 (65.1)	25 (62.5)	22 (56.4)		
2-Evening chronotype	17 (19.8)	11 (27.5)	12 (30.8)		
3-Morning chronotype	13 (15.1)	4 (10.0)	5 (12.8)		

NDD/A-ADHD = Non-Dual Disorder A-ADHD; DD/A-ADHD = Dual Disorder A-ADHD; CAARS = Conner’s Adult ADHD Rating Scales; DERS = Difficulties in Emotion Regulation Scale; RIPOST = Reactivity Intensity Polarity Stability Questionnaire; BIS = Barratt Impulsiveness Scale; BPRS = Brief Psychiatric rating scale; WHODAS = World Health Organization Disability Assessment Schedule; DIVA = Diagnostic Interview for ADHD in adults; MEQ = Morningness-Eveningness Questionnaire. “a” and “b” letters denotes a subset of categories whose column proportions differ significantly from each other at the 0.05 level.

## Data Availability

The data are not publicly available due to the privacy reasons.
